# Neuroregulation of the Hypothalamus-Pituitary-Adrenal (HPA) Axis in Humans: Effects of GABA-, Mineralocorticoid-, and GH-Secretagogue-Receptor Modulation

**DOI:** 10.1100/tsw.2006.09

**Published:** 2006-01-17

**Authors:** Roberta Giordano, Micaela Pellegrino, Andreea Picu, Lorenza Bonelli, Marcella Balbo, Rita Berardelli, Fabio Lanfranco, Ezio Ghigo, Emanuela Arvat

**Affiliations:** Division of Endocrinology and Metabolism, Department of Internal Medicine, University of Turin, Italy

**Keywords:** ACTH, cortisol, feedback, benzodiazepines, GHS, ghrelin, humans

## Abstract

The hypothalamus-pituitary-adrenal (HPA) axis exerts a variety of effects at both the central and peripheral level. Its activity is mainly regulated by CRH, AVP, and the glucocorticoid-mediated feedback action. Moreover, many neurotransmitters and neuropeptides influence HPA axis activity by acting at the hypothalamic and/or suprahypothalamic level. Among them, GABA and Growth Hormone Secretagogues (GHS)/GHS-receptor systems have been shown to exert a clear inhibitory and stimulatory effect, respectively, on corticotroph secretion. Alprazolam (ALP), a GABA-A receptor agonist, shows the most marked inhibitory effect on both spontaneous and stimulated HPA axis activity, in agreement with its peculiar efficacy in panic disorders and depression where an HPA axis hyperactivation is generally present. Ghrelin and synthetic GHS possess a marked ACTH/cortisol-releasing effect in humans and the ghrelin/GHS-R system is probably involved in the modulation of the HPA response to stress and nutritional/metabolic variations. The glucocorticoid-mediated negative feedback action is mediated by both glucocorticoid (GR) and mineralocorticoid (MR) receptors activation at the central level, mainly in the hippocampus. In agreement with animal studies, MRs seem to play a crucial role in the maintenance of the circadian ACTH and cortisol rhythm, through the modulation of CRH and AVP release. GABA agonists (mainly ALP), ghrelin, as well as MR agonists/antagonists, may represent good tools to explore the activity of the HPA axis in both physiological conditions and pathological states characterized by an impaired control of the corticotroph function.

## INTRODUCTION

The hypothalamus-pituitary-adrenal (HPA) axis is a neuroendocrine system exerting a variety of effects at both the central and peripheral level, through the action of glucocorticoids[1,2].

The activity of the HPA axis is regulated mainly by the hypothalamic CRH and AVP neurohormones which are, in turn, under the influence of several neurotransmitters and neuropeptides[1,2]; on the other hand, the glucocorticoid-mediated feedback mechanism, which takes place mainly at the pituitaric, hypothalamic, and hippocampal levels, is the most important influence integrating the HPA axis circadian activity and adapting the HPA response to stress[1,3,4,5].

Alterations in the balance of central activating and inhibiting neurotransmission on the HPA axis might be involved in the pathophysiology of some diseases such as psychiatric disorders, anorexia, and obesity, characterized by a functional hyperactivity of this axis[6,7].

This paper aims to review the present knowledge about some new aspects of the neuroendocrine control of the HPA axis in humans, in both physiological and some pathological conditions.

## GABA-BENZODIAZEPINES AND HPA AXIS (FIG. 1)

Benzodiazepines (BDZs) are one of the most prescribed classes of drugs because of their marked anxiolytic, anticonvulsant, muscle relaxant, and hypnotic effects[8]. It has been shown that many of the pharmacological actions of BDZs are mediated by specific binding sites, distributed not only in the central nervous system (CNS), but also in peripheral tissues[9]. In the CNS, BDZs bind a specific BDZ binding site that is a component of the γ-aminobutyric acid (GABA)-A receptor[9], whose activation facilitates brain GABAergic neurotransmission[10] that exerts an inhibitory influence in the CNS[8]. Moreover, GABA has been found to modulate the pituitary function by acting at the hypothalamic level, where it influences hypophysiotropic neurohormone release, and/or directly on the pituitary gland, where different GABA receptor subtypes have been detected[11]. On the other hand, a different class of BDZ receptor named "peripheral benzodiazepine receptors" (PBR) has been shown in peripheral tissues, but also in the CNS in both neurones and glial cells[9,12].

It has been clearly demonstrated that BDzs administration affects the activity of the HPA axis both in animals and in humans. The first reports, generally obtained by studying the effects of diazepam, showed conflicting results in both animals and humans. An inhibitory effect of diazepam on ACTH, corticosterone/cortisol release was reported by some authors, but not by others, in animal and human studies, while others showed no significant effects[13,14,15]. A clear evidence of the inhibitory effect of BDZs on the HPA axis came from the studies with alprazolam (ALP), a triazolobenzodiazepine that mainly acts as a GABA/BDZ receptor complex agonist[16]. ALP possesses the most remarkable inhibitory effect on the HPA axis, being 40 times more potent than diazepam, and this peculiar activity seems to explain its clinical efficacy not only as an anxiolitic agent, but also in the treatment of panic and depressive disorders in which a central HPA axis hyperactivation has been demonstrated[17].

ALP has been demonstrated to decrease urinary free cortisol significantly as well as circulating ACTH/cortisol levels in humans, enhancing the spontaneous decline of HPA activity in the morning hours, indicating a clear influence of BDZs on the basal HPA activity[18,19]. Moreover, ALP has been shown to inhibit the ACTH and/or cortisol response significantly to several stimulations such as metabolic and mental stress, naloxone, and AVP[18], while it totally abolished the ACTH response to hexarelin, a synthetic Growth Hormone Secretagogue (GHS) with ACTH-releasing effect ([20], see below). Interestingly, ALP did show a marked inhibitory effect even on ACTH rise induced by metyrapone or insulin-induced hypoglycemia, the most potent stimulation of corticotroph secretion[18,20,21], clearly indicating a primary role of BDZs in the modulation of corticotroph function. The evidence that ALP possesses a more marked inhibitory effect on the HPA axis than other BDZs is likely to reflect its high affinity for GABA/BDZ binding sites[9,10].

GABA as well as BDZs modulate the HPA axis function through central mechanisms, involving the hypothalamic control. ALP as well as diazepam, although with a lesser potency, suppress CRH secretion in the hypothalamus and locus coeruleus while neither ALP nor GABA modify basal or CRH-stimulated ACTH release in animals as wells as in humans[18,22,23].

BDZs and GABA have also been proposed to influence the HPA axis by acting at the suprahypothalamic level. ALP pretreatment significantly decreases the hippocampal CRH receptor numbers in rats[24]; moreover, in humans, it abolishes the nocturnal ACTH and cortisol rise induced by potassium canrenoate, an antagonist of both peripheral and hippocampal mineralocorticoid receptors (MR) ([25], see below). As the hippocampal MR activation is deeply involved in the glucocorticoid-mediated negative feedback mechanism, these results indicated the involvement of BDZs in the modulation of the negative feedback mechanism induced by glucocorticoids. These findings agree with the ability of ALP to inhibit the stimulatory effect of the metyrapone-induced removal of corticosteroid feedback on corticotroph secretion[19].

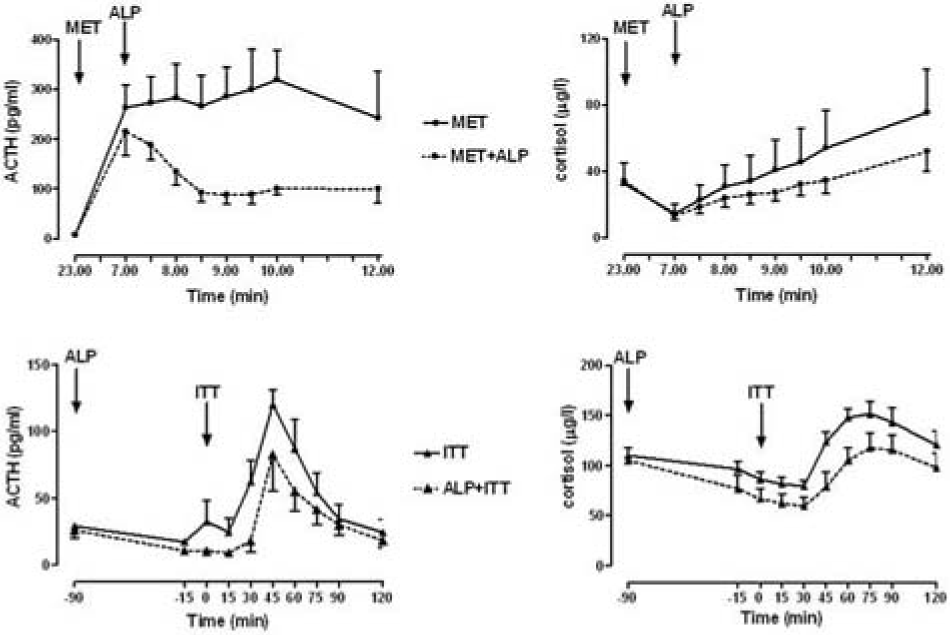

**FIGURE 1.** ACTH and cortisol levels (mean ± SEM) after metyrapone (MET, 0.04 g/kg p.o. at 2300 h on the previous night), MET + alprazolam (ALP, 0.02 mg/kg p.o. at –90 min), ITT (regular insulin, 0.1 U/kg i.v. at 0 min), or ITT + ALP in normal subjects.

Finally, although BDZs failed to modify CRH-induced ACTH secretion from rat pituitary[22], the existence of GABA/BDZ receptors in the pituitary gland[9] suggested a direct modulatory role of GABA and/or BDZs on corticotroph secretion induced by ACTH secretagogues other than CRH.

As far as the influence of PBR on the activity of HPA axis is concerned, their localization in the CNS, pituitary, and adrenal gland suggested an action of these receptors at different levels[9,12]. PBR agonists have been shown to increase CRH and ACTH release from rat hypothalamus and pituitary, respectively[26]. On the other hand, although PBR activation by endozepines stimulates steroidogenesis in the adrenal gland[12], an inhibitory effect of diazepam as well as ALP on ACTH-induced steroidogenesis in both animals and humans has been shown[12,23]. Thus, these data show how complex the effects of PBR activation on HPA axis are and suggest that PBR activation has different effects depending on different receptor localization and/or different ligands.

From the above-mentioned data, it can be assumed that BDzs are a useful tool to investigate GABAergic control of HPA function; thus, their effects have also been studied in some pathophysiological conditions associated with a disrupted control of HPA activity. It has been demonstrated that BDZs do not modify ACTH hypersecretion in patients with Cushing's disease[27], suggesting that GABA is unable to influence the corticotroph secretion in this condition. Moreover, we have previously shown that ALP possesses only a slight blunting effect on ACTH and cortisol response to hexarelin in obesity, in which HPA hyperactivation has been demonstrated[29]. The impaired inhibitory influence of BDZ/GABA on corticotroph secretion in obesity could, at least partially, contribute to the HPA hyperfunction[6].

Finally, from a clinical point of view, the above-reported data clearly indicate that the effects of BDZs on HPA function should be taken into account when endocrinological assessment in patients with suspected impaired HPA axis activity is performed; moreover, when BDZ treatment in patients with anxiogenic disorders has to be initiated, caution in the use of these drugs, especially ALP, should be used in patients with suspected hypoadrenalism.

## MINERALOCORTICOID RECEPTORS AND HPA AXIS (FIG. 2)

Glucocorticoid actions are mediated by two receptor types: type 1, or mineralocorticoid receptor (MR), and type 2, or glucocorticoid receptor (GR), both of which have been cloned in humans[3,4,28]. Animal studies showed that while GRs are distributed throughout the brain, mostly in hypothalamic neurons and corticotroph cells[3,4,28], MRs are present in the hypothalamus and highest expression has been detected in the hippocampus[3,28]. At the hippocampal level, MRs have lost aldosterone selectivity and bind glucocorticoids tenfold more than GRs; thus, differently from GRs, MRs are mostly occupied at low glucocorticoid concentrations[3,4,28].

Animal data indicate that hippocampal MRs play a major role in the control of the "proactive" feedback of glucocorticoids, devoted to the maintenance of basal HPA activity, through tonic inhibitory projections to the paraventricular nucleus[3,4,28]. On the other hand, the progressive occupancy of GRs following the increase of glucocorticoid concentrations seems to mediate, in coordinated manner with MRs, the "reactive" feedback aimed at controlling the HPA response to stress, in particular at terminating the stress-induced HPA hyperactivation[3,5,28].

A stimulatory effect of MR blockade by MR antagonists on the HPA axis has been demonstrated in both animals and humans, though controversial results have been reported in humans. In fact, a stimulatory effect of spironolactone and canrenoate on HPA axis activity has been shown by some authors, while others did not find any significant effect[29,30]. On the other hand, we have recently shown that the systemic administration of canrenoate enhances spontaneous ACTH, cortisol, and DHEA levels during the night hours, making them overlap those in morning hours, thus indicating a crucial role of these receptors in the control of HPA function at the nadir period of circadian rhythm[31].

Animal studies indicate that the hippocampus deeply influences hypothalamic CRH and AVP release, as a part of the glucocorticoid-mediated feedback mechanism[3]. The evidence that canrenoate also significantly amplified hCRH- and AVP-stimulated ACTH, cortisol, and DHEA secretion in normal individuals agrees with the hypothesis that the stimulatory effect of canrenoate on corticotroph secretion is likely to be mediated by both CRH and AVP release[31].

This hypothesis is also supported by an increased cortisol response to a physical exercise after canrenoate pretreatment, as exercise modulates HPA axis acitivity by regulation of hypothalamic control[32].

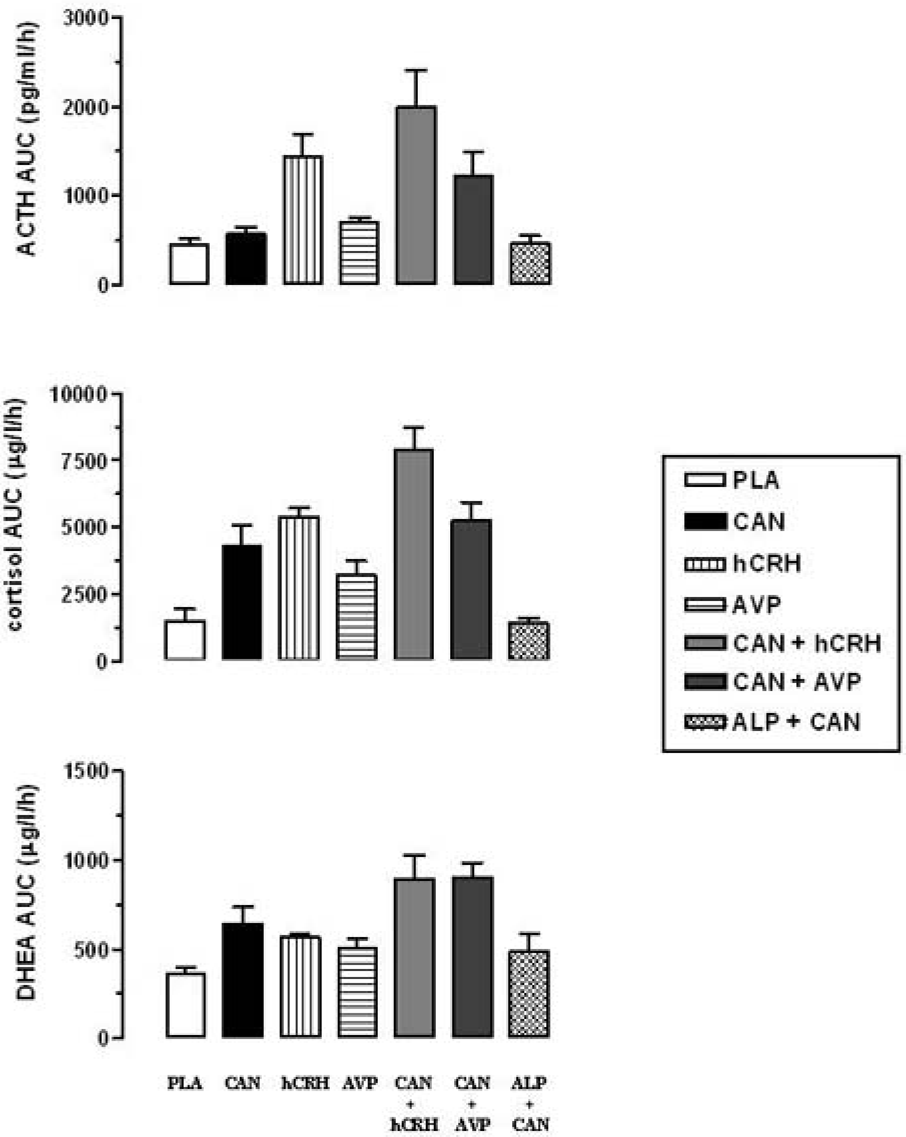

**FIGURE 2.** ACTH, cortisol, and DHEA responses (AUCs, mean ± SEM) to placebo, canrenoate (CAN, 200 mg as i.v. bolus + 200 mg as i.v. infusion from 2000–2400 h), hCRH (2.0 µg/kg i.v. at 0 min), AVP (0.17 U/kg i.m. at 0 min), CAN + hCRH, CAN + AVP, or CAN + alprazolam (ALP, 0.02 mg/kg p.o. at –90 min) in normal subjects.

The study of the mineralocorticoid antagonists effects on corticotroph and adrenal secretion represents a new approach to investigate the glucocorticoid feedback action at a suprapituitary level[33]. HPA axis hyperactivity with a disrupted circadian rhythm has been clearly demonstrated in animal and human aging[34,35] and it could be involved in the structural, metabolic, and cognitive alterations associated with aging[34,35]. HPA axis hyperactivity in aging is likely to reflect loss of resiliency and reduced sensitivity to the negative glucocorticoid feedback, which mainly reflects hippocampal receptor damage[34,35,36]. Human studies have, so far, generally been performed by studying the HPA sensitivity to the negative feedback action of dexamethasone, which does not easily cross the blood brain barrier, and/or of pharmacological hydrocortisone doses[37,38]. Thus, these experimental models would not conceptually represent the most reliable way to evaluate the age-dependent variations in the feedback control of the HPA axis, which is mainly under central modulation. We have recently shown elevated ACTH and cortisol levels during the night hours in elderly subjects, which are only slightly increased by canrenoate administration. In agreement with animal data, these findings indicate an impairment of MR function in human aging[39]. Surprisingly, despite the well-known age-related reduction in DHEA synthesis and secretion, probably reflecting particular atrophy of the reticularis zone of the aged adrenal gland[40,41], canrenoate significantly increased nocturnal DHEA levels in the elderly, suggesting a peculiar preserved stimulatory effect of MR antagonism on reticularis zone in aging[39].

An impairment of central MR function is also involved in the occurrence of HPA hyperactivity generally observed in major depression. Reduced MR density has been shown in the hippocampus of depressed subjects from autoptic studies[42]. Interestingly, many antidepressant agents are able to increase GR and MR expression in several CNS areas, including the hippocampus, and this is associated with an improvement in the clinical feature as well as HPA activity in depressed subjects after pharmacological treatment[42]. These data clearly indicate the involvement of HPA dysfunction in the occurrence of the clinical symptoms of depression and suggest that the clinical efficacy of antidepressant drugs may, at least partially, involve the improvement of the glucocorticoid-mediated inhibitory feedback action on HPA activity.

## GHRELIN, SYNTHETIC GH SECRETAGOGUES (GHS), AND HPA AXIS (FIG. 3)

Ghrelin, a 28 aminoacid peptide predominantly produced by the stomach, a natural ligand of the orphan GH secretagogue receptor type 1a (GHS-R1a), is known to play a major role in the control of food intake and energy balance, although it also exerts important central and peripheral neuroendocrine and nonendocrine actions, including a strong GH-releasing effect as well as a positive influence on lactotroph and corticotroph secretion[43,44,45,46].

GHS-R are expressed in the hypothalamus-pituitary unit, but also in other areas of the CNS and in peripheral tissues, particularly in the adrenal gland[47,48].

The stimulatory effect of ghrelin and GHS on the activity of the HPA axis has been clearly demonstrated both in animals and in humans[43,44,45,46]. The increase in ACTH and cortisol levels has been demonstrated in man after acute administration of both ghrelin and GHS[43,44,45,46]. Interestingly, the maximal stimulatory dose of GHS on the HPA axis is lower than that needed for the maximal GH-releasing effect[45,46]. The stimulatory effect of GHS on the HPA axis is not negligible, overlapping that after AVP or naloxone, and being even similar to that after hCRH[44,45]. This seems, however, an acute neuroendocrine effect; in fact, during prolonged GHS treatment, no variations in 24-h cortisol profile have been found[45].

The ACTH-stimulatory effect of ghrelin and GHS is gender independent, but dependent on BMI. In fact, ACTH and cortisol responses to ghrelin have been shown increased and reduced, respectively, in obesity and anorexia nervosa, despite low and high total ghrelin levels in obese and anorectic patients, respectively[49,50]. This suggests some alterations in the HPA sensitivity to ghrelin administration in these pathological conditions.

On the other hand, some peculiar age-related variations in the corticotroph-stimulatory action of GHS are present. In fact, the ACTH response to hexarelin, a synthetic GHS, increases at puberty, then shows clear reduction in adulthood and, finally, a new trend toward increase in aging[51]. The increased effect at puberty could depend on estrogens, while the rebounded effect in aging agrees with evidence showing HPA hyperactivity due to neuroendocrine changes in the aged brain[35,51].

The mechanisms underlying the ACTH/cortisol-releasing effect of ghrelin and GHS seem to be mediated via central actions, at least in physiological conditions. In fact, GHS are unable to stimulate ACTH secretion from both animal and human pituitary cells in culture; moreover, the ACTH-releasing activity of GHS is abolished after hypothalamo-pituitary disconnection[44,45,46]. Animal and human data indicate CRH and/or AVP-mediated mechanisms of action: both GHS and ghrelin increase CRH and AVP output from the hypothalamus, while in humans the coadministration of hexarelin and hCRH, as well as AVP, does not show additive or synergistic effect[44,45,46]. Interestingly, the central actions of ghrelin also include an anxiogenic effect that is abolished by pretreatment with a CRH antagonist in rodents, thus pointing out the involvement of CRH secretion in the central neuroendocrine and nonendocrine actions of the peptide[52]. The possibility that the ACTH-releasing activity of GHS could be mediated by other neural pathways has also been investigated. The ACTH-releasing activity of hexarelin is not mediated by histaminergic or serotoninergic pathways, which play a critical role in the neural control of the HPA axis[2,53], while it seems to be influenced by GABA (see above). Moreover, there is evidence that the majority of hypothalamic neurons activated by ghrelin are NPY positive[44,45]. As the key role of this neuropeptide in the control of the HPA axis is widely accepted, NPY may be another possible candidate to mediate the ACTH-releasing effect of ghrelin[45].

The ACTH-releasing activity of both ghrelin and GHS is sensitive to the negative glucocorticoid-mediated feedback mechanism in physiological conditions. In fact, it is potentiated after metyrapone in normal subjects as well as in patients with Addison’s disease[54]. On the other hand, it is inhibited by acute or chronic glucocorticoid administration as well as in patients with Cushing’s syndrome bearing a cortisol-secreting adrenal tumor[20,55,56]. It is remarkable that, despite hypercortisolism, both ghrelin and synthetic GHS display marked increase in their stimulatory effect on ACTH and cortisol secretion in patients with pituitary ACTH-dependent Cushing’s disease[55,56,57,58]. The stimulatory effect of ghrelin and GHS on corticotroph secretion is even surprisingly higher than that of hCRH and generally more marked in patients bearing ACTH-secreting microadenoma[55,56]. The exaggerated ACTH and cortisol response to GHS in Cushing’s disease is intriguing considering that, meantime, the GH response to ghrelin and GHS is markedly reduced by glucocorticoid excess[55,56,57,58]. This is likely to reflect a direct action on the GHS-R expressed by the neoplastic cells. In fact, the existence of GHS-R in several neuroendocrine tumors, including both pituitaric and ectopic ACTH-secreting tumors, has been clearly demonstrated[59]. This GHS-R distribution also explains the peculiar ACTH hyperresponsiveness to ghrelin and GHS even in ectopic ACTH-secreting tumors[60]. These observations, however, reduce the potential use of GHS in testing ACTH secretion to distinguish patients with pituitary from ectopic ACTH-dependent hypercortisolism.

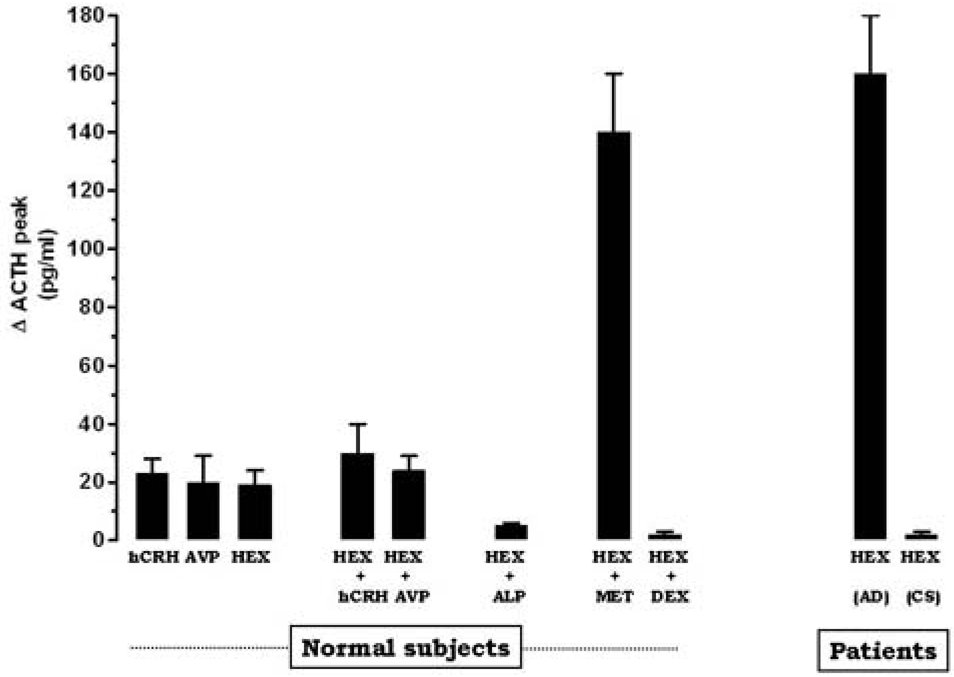

**FIGURE 3.** ACTH responses (Δpeak, mean ± SEM) to hCRH (2.0 µg/kg i.v. at 0 min), AVP (0.17 U/kg i.m. at 0 min), hexarelin (HEX, 2.0 µg/kg i.v. at 0 min), HEX + hCRH, HEX + AVP, HEX + alprazolam (ALP, 0.02 mg/kg p.o. at –90 min), HEX + metyrapone (MET, 0.04 g/kg p.o. at 2300 h on the previous night), HEX + dexamethasone (DEX, 1 mg p.o. at 2300 h on the previous night) in normal subjects, and to HEX in patients with Addison’s disease (AD) and Cushing’s disease (CD).

Finally, it has to be taken into account that the adrenal gland is probably the tissue with the highest expression of GHS receptors, while ghrelin mRNA in the adrenals has been shown by some authors, but not others[46,47]. The functional role of ghrelin at the adrenal level is still unknown; in fact, ghrelin does not play any role in adrenal steroidogenesis, but it could modulate adrenal cell viability in both physiological and neoplastic conditions[46].

## CONCLUSIONS

HPA is a neuroendocrine axis with a wide range of actions mainly devoted to integrate behavioral and metabolic responses to stress. Thus, it not surprising that its control is exerted by a complex network ranging from neural pathways, which modulate behavior and anxiety, to factors involved in energy balance regulation.

GABA and BDZs exert an inhibitory role and ALP showed the most marked inhibitory effect on both spontaneous and stimulated HPA axis activity, in agreement with its peculiar efficacy in panic disorders and depression, where an HPA hyperactivation is generally present. For these reasons, BDZs, especially ALP, may be good tools to study the HPA axis neuroregulation. Moreover, the effects of these substances have to be taken into account when endocrinological investigations are performed, as well as before treating patients with suspected adrenal impairment.

Glucocorticoid-mediated feedback action is the most important peripheral mechanism controlling HPA axis activity via central GR and MR activation. The chronic HPA hyperactivation, which connotes several pathophysiological conditions, is likely to reflect impairment of central MRs impinging on the HPA axis. Recent evidence indicates that studying the effect of MR antagonists on corticotroph and adrenal secretion represents a new and useful approach to investigate the glucocorticoid feedback effect at a suprapituitary level in humans.

Finally, the ghrelin/GHS system is probably involved in the modulation of the HPA response to stress and nutritional/metabolic variations, but also in the modulation of corticotroph and adrenal cell viability.

## ACKNOWLEDGMENTS

The authors wish to thank Prof. Franco Camanni for his participation in the studies mentioned in this paper and Dr. Angela Bertagna, Mrs. Anna Barberis, and Marina Taliano for their skillful technical assistance.
